# Haematopoietic Stem Cell Transplant for Norovirus-Induced Intestinal Failure in X-linked Agammaglobulinemia

**DOI:** 10.1007/s10875-021-01088-2

**Published:** 2021-06-23

**Authors:** Ben M. J. Shillitoe, Mark Ponsford, Mary A. Slatter, Jennifer Evans, Siske Struik, Mike Cosgrove, Iolo Doull, Stephen Jolles, Andrew R. Gennery

**Affiliations:** 1 Paediatric Immunology, Newcastle Upon Tyne Hospital Trusts, Great North Children’s Hospital, Clinical Resource Building, Floor 4, Block 2, Queen Victoria Road, Newcastle upon Tyne, NE1 4LP UK; 2 Immunodeficiency Centre for Wales, Cardiff and Vale University Health Board, University Hospital Wales, Cardiff, UK; 3 Translational and Clinical Research Institute, Newcastle Upon Tyne University, Newcastle upon Tyne, UK; 4 Department of Paediatric Gastroenterology, Singleton Hospital, Swansea, Wales UK; 5 Department of Paediatric Respiratory Medicine, Cardiff and Vale University Health Board, University Hospital Wales, Cardiff, UK

**Keywords:** X-linked agammaglobulinemia, haematopoietic stem cell transplantation, norovirus, inflammatory bowel disease

## Abstract

Since the first clinical description in 1952, immunoglobulin replacement therapy remains the mainstay of treatment of patients with X-linked agammaglobulinemia (XLA). However, this therapy only replaces IgG isotype and does not compensate for the loss of Bruton tyrosine kinase in non-B-lymphocytes. Patients may still therefore develop complications despite current standard of care. Here, we describe an XLA patient with persistent chronic norovirus infection, refractory to treatment and causing intestinal failure. The patient underwent haematopoietic stem cell transplantation, curing XLA and allowed clearance of norovirus prior to humoral immunoreconstitution, suggesting non-humoral immunodeficiency in these patients.

## Introduction

X-linked agammaglobulinemia (XLA) is caused by defects in the gene encoding Bruton’s tyrosine kinase (BTK), characterised by the absence of peripheral circulating CD19 + B-lymphocytes and agammaglobulinemia. XLA typically presents with recurrent infections, usually by encapsulated bacteria after 6 months of age, as maternally transferred antibodies wane [1].

The mainstay of treatment is lifelong immunoglobulin replacement therapy (IgRT) and has been since XLA was first described by Colonel Ogden Bruton in 1952 [1]. There have been substantial improvements in manufacturing, tolerability, and flexibility in routes of administration of IgRT seen in recent decades [2, 3]. Unlike other primary immunodeficiencies, haematopoietic stem cell transplantation (HSCT) is not routinely offered for XLA patients, as IgRT is accepted as an effective therapy, and it is generally believed that the benefits of HSCT do not outweigh the risks.

However, as IgRT contains only the IgG isotype, XLA patients receiving treatment remain IgM and IgA deficient. In addition, BTK is expressed in all myeloid cells and may play important immune functions beyond B-lymphocytes [4]. This is supported by the presentation of XLA patients with infections other than encapsulated infection such as giardia and enterovirus [5, 6]. In addition, up to 26% of patients present with neutropenia and infections with pseudomonas [7]. Neutropenia and subsequent pseudomonal infection are rare once IgRT has been commenced, suggesting that the neutropenia is somehow IgG dependant, although the exact mechanism by which this occurs is unknown [8]. The failure to compensate for the loss of IgA, IgM, and for the other functions of BTK implies that XLA patients may still be susceptible to complications.

This is illustrated by the increased risk of development of chronic lung disease (CLD) due to bacterial infections demonstrated by Lougaris et al., calculating the risk of developing CLD at 50 years of age of 47% [9].

Furthermore, there is gathering evidence of the impact from viral infections in XLA patients, and antibody deficiency more generally [6, 10]. Taken together, these findings suggest that the impact of the burden of bacterial and viral infection on morbidity and mortality has been significantly underestimated in XLA.

Chronic, treatment-refractory norovirus infection in common variable immunodeficiency (CVID) is well documented [11]. There is one report of chronic norovirus in an XLA patient, which was unresponsive to treatment and the patient continues with chronic norovirus infection [12]. We describe an XLA patient with persistent severe chronic norovirus-associated enteropathy resulting in intestinal failure and requiring parental nutrition (PN), for whom HSCT was performed to offer cure of XLA and clearance of norovirus infection.

## Methods

Clinical data were collated from the printed and electronic medical record. Immunoglobulin levels (IgG, IgA, and IgM) were assayed by nephelometry (Siemens BN2 Nephelometer; Siemens), and serum electrophoresis (Sebia Capillarys 2; Sebia, Norcross, GA, USA). Where immunoglobulin was detectable, the lower limit of assay sensitivity (IgG, 1.34 g/L; IgA, 0.24 g/L; and IgM, 0.17 g/L) was used for data analysis. Specific antibody titres against haemophilus influenzae, tetanus, and pneumococcal capsular polysaccharide were determined by enzyme-linked immunosorbent assay (The Binding Site, Birmingham, UK). All testing was performed in the United Kingdom Accreditation Service accredited Immunology Laboratory at the University Hospital of Wales. Lymphocyte subsets were enumerated by flow cytometry. Donor chimerism was measured using short tandem repeats, in whole blood, or in separated myeloid, B-lymphocyte and T-lymphocyte lineages. All procedures were performed after informed consent was given by the parents, according to institutional protocols.

## Results

**Fig. 1:**
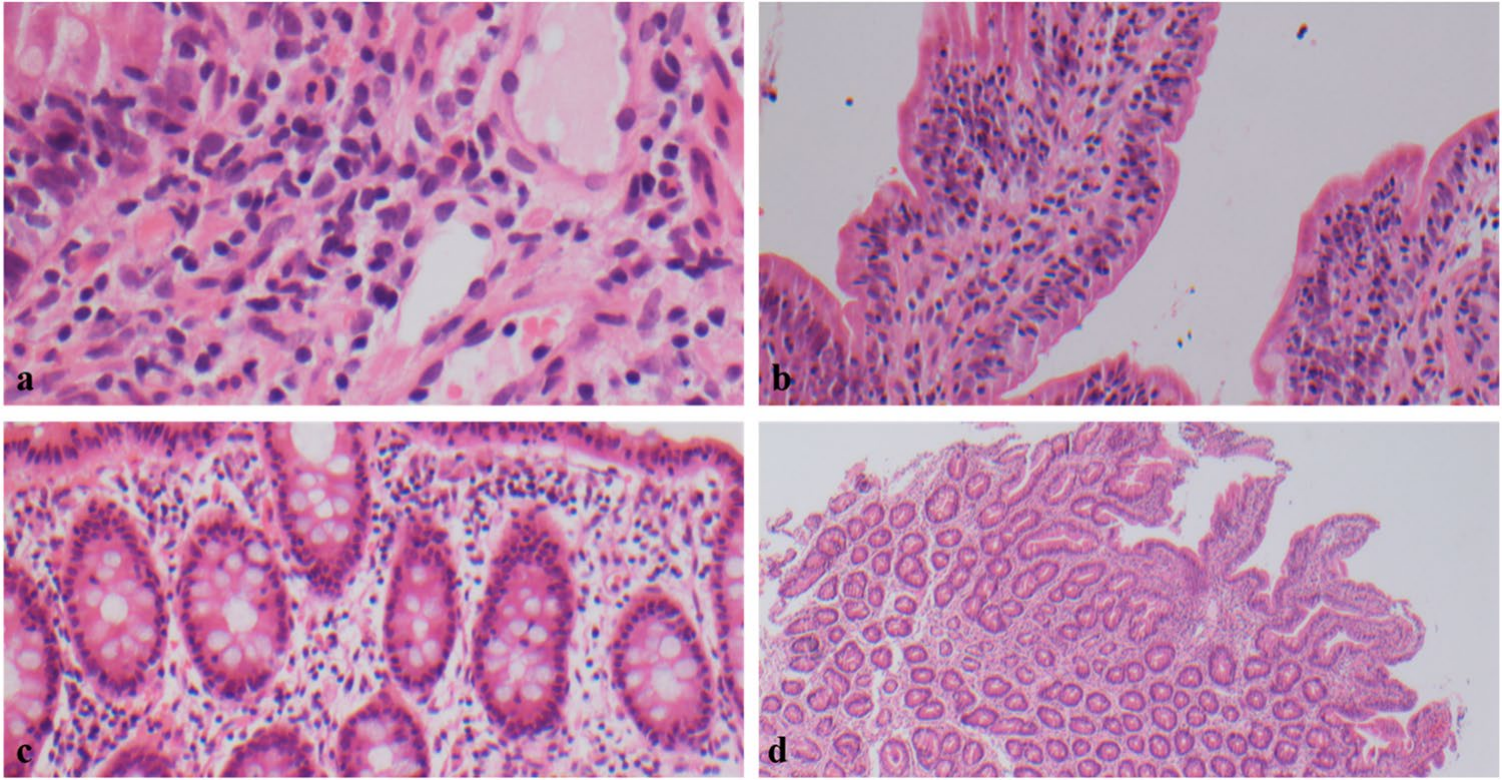
Histology of small and large bowel (courtesy of Dr Namor Wyn Williams). **a** Absence of plasma cells within lamina propria. **b** Intraepithelial lymphocytes within duodenum. **c** Increase of intraepithelial lymphocytes within large bowel. **d** Villous blunting within duodenum

The first male child of non-consanguineous Caucasian parents presented with varicella at age 8 weeks, followed by 2 episodes of zoster. Hypogammaglobulinaemia was noted on his second admission, associated with neutropenia. A diagnosis of XLA was made aged 8 months based on absent B-lymphocytes, and low monocyte BTK expression. T-lymphocyte subsets and PHA proliferation studies were normal. Sanger sequencing confirmed a previously reported c.1750 + 2 T > C pathogenic variant in *BTK* [13]. IgRT was commenced with intravenous immunoglobulin (IVIg) loading and maintenance subcutaneous infusions (SCIg) at 1 year at 0.1–0.2 g/kg/week. In the first 2 years of life, he was well, other than mild, recurrent *Haemophilus influenzae* conjunctivitis. Between the ages of 3 and 6 years, he required 4 courses of intravenous broad-spectrum antibiotics for respiratory infections. His mean IgG trough level was 6.7 g/L. The intracellular pathogen, *Mycobacterium abscessus*, was repeatedly cultured on induced sputum samples, and bronchoalveolar lavage (BAL) isolated *Haemophilus influenza*. Thoracic computerised tomography (CT) showed no bronchiectasis. Treatment with 3 weeks of intravenous cefoxitin, imipenem, amikacin, with oral co-trimoxazole, and clarithromycin was successful in eradicating *Mycobacterium abscessus*. At the age of 5 and a half years of age, faltering growth was noted associated with increased stool frequency, urgency, and nocturnal disturbance. An empirical course of high-dose metronidazole failed to ameliorate symptoms. Coeliac risk-associated HLA DQ2 or DQ8 alleles were absent. Faecal calprotectin was elevated (226.3 and 583.6 μg/g; normal level < 100 μg/g) and abdominal ultrasound showed three separate areas of small bowel intussusception, despite remaining pain-free. At this time, no pathogens were isolated in the stool and the intussusception resolved without any intervention. Upper and lower gastrointestinal tract endoscopy showed no gross abnormalities. Duodenal biopsy showed evidence of chronic enteropathy with villous blunting and increased intraepithelial lymphocytes (Fig. 1).

**Fig. 2:**
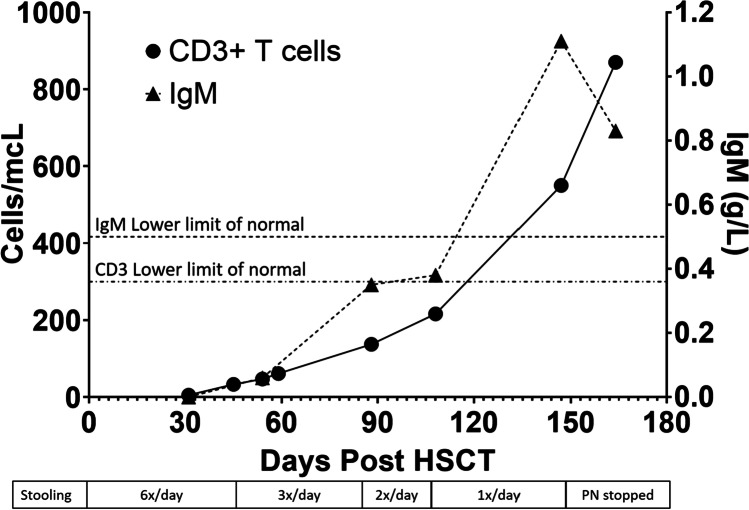
Immune reconstitution post-HSCT and relation to GI symptoms for the first 12 months post-HSCT

**Fig. 3:**
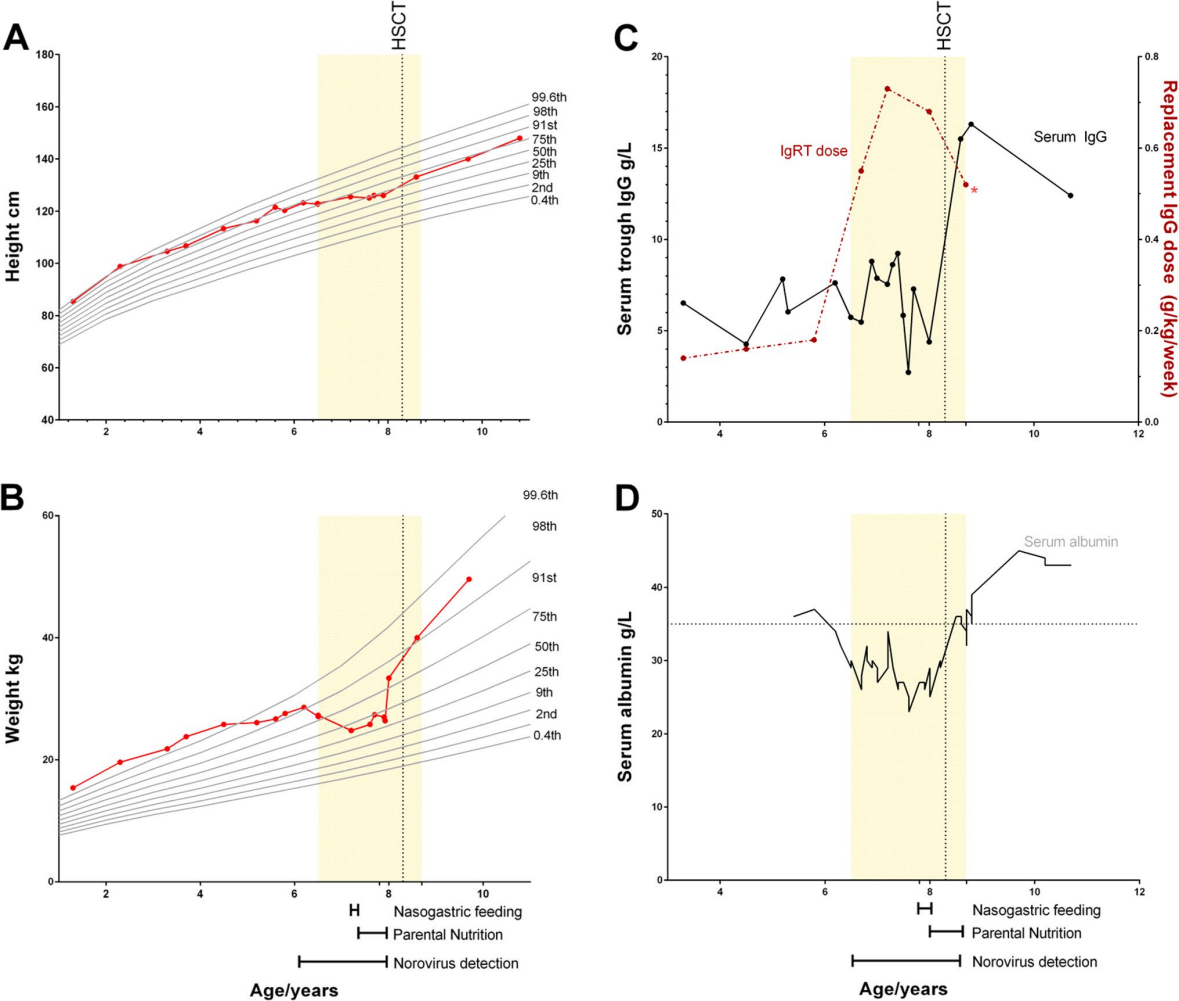
Growth, biochemical, and treatment trends. Shaded area represents the time period when the patient was positive for type 2 norovirus in stools. Vertical dotted line indicates timing of haematopoietic stem cell transplant (HSCT). **A** Height and **B** weight trends are plotted relative to UK reference centiles. **C** Serum IgG trough levels (left y-axis) with immunoglobulin replacement therapy (IgRT) dose (dashed line, right y-axis). **D** Serum albumin. Dashed line indicates the lower limit of normal (35 g/L)

Extended stool microbiology testing identified type 2 norovirus, but was negative for all other pathogens, including *Giardia lamblia* and *Campylobacter jejuni* (Fig. 2). During this time, his oral intake became restricted, he experienced severe nausea and bloating, and his quality of life significantly decreased. Despite dietary supplementation including nasogastric (NG) feeding, his growth faltered, falling from the 99.6th centile for weight and height aged 6.2 years to the 58th centile for weight and 41st for height by age 8.0 years **(**Fig. 3A, B. He subsequently failed to gain further weight, and serum albumin at this time was 27 g/L, associated with peripheral oedema (Fig. 3D). Serum trough IgG levels fell to 2.73 g/L despite escalating subcutaneous replacement doses in line with body weight. In order to maintain an adequate trough level exceeding 6 g/L, 3 weekly intravenous immunoglobulin infusions were instigated alongside weekly SCIg, equating to a weekly IgRT dose of 0.5–0.7 g/kg/week (Fig. 3C). Bovine colostrum, oral ribavirin, and modified release oral budesonide were trialled; however, norovirus and gastrointestinal symptoms persisted. Age 8 years, acute-on-chronic flares of nausea, vomiting, and diarrhoea prompted instigation of PN. This allowed modest, if intermittent, weight gain over the next 6 months. In view of the faltering growth and treatment-resistant intestinal failure associated with chronic norovirus infection in the setting of primary immunodeficiency, HSCT was offered.

Aged 8 years old, the patient underwent a 10/10 HLA-matched unrelated peripheral blood stem cell transplant (CD34 + dose 9.0 × 10^6^/kg). Conditioning consisted of fludarabine 150 mg/m^2^, treosulfan 42 g/m^2^, thiotepa 10 mg/kg, and alemtuzumab 1 mg/kg. Graft-versus-host disease (GvHD) prophylaxis consisted of mycophenolate mofetil until day + 42 and ciclosporin.

Pre-transplant, *Streptococcus pneumoniae* was detected on routine bronchoalveolar lavage and treated with IV co-amoxiclav. *Campylobacter jejuni* was detected in stools and treated with azithromycin from day − 4. His stools were negative for *Campylobacter jejuni* by day + 3.

He developed moderate acute kidney injury (eGFR 54 ml/min/1.73 m^2^), which self-resolved within the first 12 months post-transplant. There were no other HSCT-related complications. He achieved neutrophil engraftment on day + 19 and platelet engraftment on day + 17. He demonstrated no evidence of GvHD. Whole blood chimerism on day + 19 was 100%. His latest chimerism, + 843 days post-HSCT, demonstrates 78% CD15 donor chimerism, 93% B-lymphocyte donor chimerism, and 71% donor T-lymphocyte chimerism.

By day + 88 post-HSCT, the patient’s stooling frequent had reduced from 6 times day pre-HSCT to twice a day and to once daily by day + 108. Norovirus became undetected by PCR on day + 147 post-HSCT, for the first time in over 19 months, and parenteral nutrition was discontinued. At this stage, his IgRT dose was reduced to 0.15 g/kg/week.

IgRT dosing was stopped at day + 224, and at last follow-up, his CD19 count is 660 cells/μL (normal range 200–1600), IgG 12.4 g/L (normal 4.9–16.1), and IgM 0.78 g/L (0.5–1.80). He demonstrated excellent vaccine antibody responses following post-transplant routine childhood vaccinations (total pneumococcal antibody titre 153 mg/L, HiB antibody > 9 mg/L, and tetanus 1.64 IU/mL). A normal enteral diet has been resumed without the need for supplementation, and the patient is thriving. Height has returned to the 75th centile for age and weight between the 91st and 99th centile. His lung function is normal (FEV1 103% predicted, FVC 102% predicted).

## Discussion

Norovirus-associated enteropathy in primary and secondary immunodeficiency is recognised as a chronic and debilitating disorder for which no reliable treatment options exist [14]. Given the clinical and biochemical evidence of intestinal failure and the risks associated with long-term parenteral nutrition, we sought to correct the underlying immunological defect. To our knowledge, this is the first reported case of chronic norovirus-associated enteropathy in X-linked agammaglobulinemia cured by HSCT, although patients with X-linked SCID, poor immune reconstitution, and chronic norovirus enteropathy have responded to autologous gene therapy [15]. It is likely that our patient had protein-losing enteropathy, likely associated with chronic infection, and a degree of inflammatory colitis is also likely, which may be associated with X-linked agammaglobulinemia. We also contribute to the growing evidence base that HSCT can correct XLA [16–24] (Table 1). Of 16 cases reported to date, there is a mix of indications including a diagnosis of XLA, in countries where patient access to IgRT is not assured, malignancy and chronic, unremitting infection. All patients reported to date are alive, and all, but one, have discontinued IgRT, even in the presence of mixed chimerism. No consistent conditioning regimen has been used, but a low toxicity myeloablative regimen without total body irradiation appears satisfactory.

**Table 1:** Summary of published data of haematopoietic stem cell transplantation for X-linked agammaglobulinemia

Author, number of patients	Patient age (years)	Indication	Conditioning	Stem cell source	GvHD	Donor chimerism	Discontinued IgRT	Outcome (FU–years)
Huang et al., 2	N/A	XLA	N/A	MSD BM	No	N/A	Y	A 2 13
Wan et al., 1	14	XLA	Anti-CD3 Bu12/Cy200	MUD UCB	No	100%	Y	A 1
Abu‐Arja et al., 1	14	AML	TBI (1.2 Gy) Cy120 Etop40	MUD PBSC	aGvHD II	100%	Y	A 2
Ikegame et al., 1	28	Infectious complications	ATG (2.5 mg) Flu180 Cy100 TBI (3 Gy)	MSD PBSC	aGvHD I	100%	Y	A 1
Van Zelm et al., 1	25	Pre-B ALL	TBI (6 Gy) Etop60	MSD PBSC	Limited cGvHD	100%	Y	A 1
Nie et al., 2	4	Infectious complications	ATG6 Flu150 Bu12 Ara-C3g/m^2^ Cy50	mMUD-PTCy	Limited cGvHD	100%	Y	A 2
	24	T cell lymphoma	ATG5 Flu80 Bu12.8 Cy50	Haplo-id sib-PTCy	No	100%	Y	A 2
Swaminathan et al., 2	1.42	XLA	TT8 Treo42 Flu160	MSD BM	cGvHD—skin	100%	Y	A 3
	1.67 (1st)	XLA	Bu12.8 Flu160	MSD BM	No	Rejected	N	A
	2.83 (2nd)		TT8 Treo42 Flu160	MSD PBSC	No	100%	Y	A 1
Rawat et al., 4	N/A	XLA	TT,Treo,Flu*	MSD	aGvHD	100%	Y	A N/A
	N/A	XLA	TT,Treo,Flu*	MSD	No	100%	Y	A N/A
	N/A	XLA	Flu,Treo*	Haplo-id parent	No	mixed	Y	A N/A
	N/A	XLA (1st)	Flu,Bu*	MSD	No	Rejected	N	
		XLA (2nd)	TT,Treo,Flu*	MSD	No	100%	Y	A N/A
Bucciol et al., 1	14	Chronic Aichi virus	Treo42 Flu120	MSD PBSC	aGvHD II Limited cGvHD	100%	N	A 2
Shillitoe et al., 1	8	Chronic Norovirus	Alemtuzumab TT10 Treo42 Flu150	MUD PBSC	No	78% CD15 93% CD19 71% CD3	Y	A 2

*ALL*, acute lymphoid leukaemia; *AML*, acute myeloid leukaemia; *Ara-C*, cytarabine; *ATG*, anti-thymocyte globulin; *BM*, bone marrow; *Bu*, busulfan; *Cy*, cyclophosphamide; *Etop*, etoposide; *Flu*, fludarabine; *a/cGvHD*, acute/chronic graft-versus-host disease; *Gy*, gray; *MSD*, matched sibling donor; *(m)MUD*, (mis)matched unrelated donor; *N/A*, not available; *PBSC*, peripheral blood stem cells; *PTCy*, post-transplant cyclophosphamide; *TBI*, total body irradiation; *Treo*, treosulfan; *TT*, thiotepa; *UCB*, umbilical cord blood; *XLA*, X-linked agammaglobulinemia; *doses not available

The mechanism by which XLA patients are susceptible to chronic norovirus infection remains unclear, but likely reflects the multiple roles of BTK in immune homeostasis. Beyond production of immunoglobulins, B-lymphocytes are potent antigen-presenting cells [25]. BTK is expressed in other immune cells, e.g., NK cells, macrophages, monocytes, neutrophils, and dendritic cells [26], where it participates in Toll-like receptor and NLRP3 inflammasome signalling [26, 27]. Loss of B-lymphocyte co-stimulation in CD4 + T-lymphocyte differentiation and impairment of types I and III interferon pathways has also been demonstrated in XLA patients [28, 29]. This is consistent with the susceptibility to a range of pathogens seen in our patient and the emerging literature that demonstrates that XLA patients are more vulnerable to frequent and chronic infections across a range of pathogens (including norovirus and mycobacterium abscessus) than has been previously appreciated [6, 12, 30].

In our patient, diarrhoea began to improve with T-lymphocyte reconstitution before any significant humoral reconstitution, although the initial reduction in stooling may have been related, at least in part to the conditioning chemotherapy, which may have effectively treated any co-existing inflammatory bowel disease. Prior to HSCT, the patient was passing 5–6 Bristol-type 6/7 stools a day. At + 47 days post-HSCT, he was passing 3 stools a day, when his CD3 count was 47 cells/μL, serum IgA was < 0.05 g/L, and serum IgM was 0.06 g/L. By day + 88 post-HSCT, his stooling frequency was twice a day, and his CD3 count was 137 cells/μL, serum IgA was < 0.05 g/L, and serum IgM was 0.35 g/L. This timing of symptom improvement with T-lymphocyte reconstitution rather than significant production of IgM/IgA lends further credence to an undefined deficit in cellular immunity pre-HSCT predisposing him to chronic norovirus infection, rather than agammaglobulinemia (noting also that prior IgRT likely contained anti-norovirus antibodies and did not clear infection).

The immunological defects associated with BTK deficiency are likely only partially corrected by current approaches to treatment and prevention of infection, which differs little from Bruton’s original prescription of immunoglobulin replacement and antibiotics. It is important to note that current IgRT products typically replace only IgG, and do not include IgA and IgM. Consistent with an incomplete protection, we have recently highlighted viral persistence in XLA and other patients with predominant antibody deficiency despite standard of care [30].

There are a number of XLA patients who experience an inflammatory bowel disease-like phenotype, some of whom are given a formal diagnosis of Crohn’s disease [31]. The pathogenesis behind this inflammatory phenotype in XLA is poorly understood. Whilst this might be a direct consequence of BTK deficiency, it is possible that this inflammation is a post-viral infection phenomenon.

Patients with XLA are at elevated risk of developing malignancy and inflammatory bowel disease, risks which are unlikely to be altered by IgRT [16, 31]. Whilst HSCT is not currently routinely considered for patients with XLA, given the availability and safety of IgRT, we believe a paradigm shift of thought may be required. For instance, Ikegame et al. have recently reported successful HSCT for an adult with XLA experiencing recurrent infections and increasing risk of end-organ damage [19]. HSCT for XLA may be appropriate as standard of care in health care settings where reliable access to IgRT is not possible, particularly in resource-poor health care settings where HSCT is more cost effective than lifelong IgRT. Across all health care settings, the cost of lifelong IgRT is considerable, whilst access remains an ongoing concern, and the potential use of immunoglobulin therapy for COVID-19 has added to those pressures [32]. Although there are no cost-effectiveness analyses available, HSCT may be more cost effective than IgRT even in developed health care settings.

The decision regarding offering HSCT for any PID is complex and the potential benefit of complete cure and improved quality of life needs to be carefully balanced against the significant complications seen in HSCT, namely infection and GvHD which are associated with significant morbidity and mortality. Whilst limited data suggest that XLA patients will experience disease-related complications despite best current therapy, this has to be confirmed in larger cohorts, and so HSCT for XLA remains controversial and novel. However, for other PIDs, such as chronic granulomatous disease, HSCT was historically considered experimental, but is now offered as routine care due to improving transplant outcomes and increased awareness of the disease complications, particularly in adults [33], and improvement in quality of life [34]. Perhaps, given the safety of HSCT in the current era, and the morbidity and decreased quality of life documented in XLA patients on lifelong antibody treatment [35], HSCT should be considered more often, particularly in younger patients, for whom transplant results are most successful.

In conclusion, this case demonstrates the curative role of HSCT for XLA alongside the complication of norovirus-associated enteropathy. It illustrates the importance of correction of cellular immune responses in this disease, and the limitations of conventional therapeutic approaches to prevent or treat illness. Continued improvement in the safety profile and outcomes for HSCT and related approaches, such as gene therapy, are likely to widen the offer of a similar curative approach to other patients with XLA in the future.

## Data Availability

For further information, please contact ARG: a.r.gennery@ncl.ac.uk.
